# Interferon Impedes an Early Step of Hepatitis Delta Virus Infection

**DOI:** 10.1371/journal.pone.0022415

**Published:** 2011-07-19

**Authors:** Ziying Han, Shoko Nogusa, Emmanuelle Nicolas, Siddharth Balachandran, John Taylor

**Affiliations:** Fox Chase Cancer Center, Philadelphia, Pennsylvania, United States of America; Yonsei University, Republic of Korea

## Abstract

Hepatitis delta virus (HDV) infects hepatocytes, the major cell type of the liver. Infection of the liver may be either transient or chronic. The prognosis for patients with chronic HDV infection is poor, with a high risk of cirrhosis and hepatocellular carcinoma. The best antiviral therapy is weekly administration for at least one year of high doses of interferon alpha. This efficacy of interferon therapy has been puzzling in that HDV replication in transfected cell lines is reported as insensitive to administration of interferon alpha or gamma. Similarly, this study shows that even when an interferon response was induced by transfection of poly(IC) into a cell line, HDV RNA accumulation was only modestly inhibited. However, when the HDV replication was initiated by infection of primary human hepatocytes, simultaneous addition of interferons alpha or gamma at 600 units/ml, a concentration comparable to that achieved in treated patients, the subsequent HDV RNA accumulation was inhibited by at least 80%. These interferon treatments were shown to produce significant time-dependent increases of host response proteins such as for Stat-1, phosphoStat-1, Mx1/2/3 and PKR, and yet interferon pretreatment of hepatocytes did not confer an increased inhibition of HDV replication over interferon treatment at the time of (or after) infection. These and other data support the interpretation that interferon action against HDV replication can occur and is largely mediated at the level of entry into primary human hepatocytes. Thus in vivo, the success of long-term interferon therapy for chronic HDV, may likewise involve blocking HDV spread by interfering with the initiation of productive infection of naïve hepatocytes.

## Introduction

Hepatitis delta virus (HDV) was initially discovered because it can induce an acute exacerbation of chronic hepatitis B virus (HBV) infections [1]. HDV is now known to be a subviral agent that requires the envelope proteins of HBV for infection of hepatocytes and for assembly of new virus particles [2]. About 75% of patients chronically infected with both HDV and HBV will develop liver damage at a significantly greater rate than patients infected with HBV alone [3].

As a preventive measure, strategies that block HBV infection also block HDV. Thus, the recombinant HBV vaccine comprised of the HBV envelope proteins is the best strategy for preventing both HBV and HDV infections. Worldwide adoption of this vaccination strategy has decreased the incidence of HBV infections, and in turn, HDV because it is dependent upon HBV as a helper virus [4], [5].

On the other hand, once HDV infections have occurred and become chronic in an HBV carrier, nucleoside analog inhibitors of HBV replication do not lead to significant decreases in HDV [4]. Currently, the best therapy for chronic HDV infection involves treatments of up to 2 years with weekly injections of high doses (9 million units) of pegylated interferon alpha [4], [6], [7] which is also active against HBV. Even then, the success rate for such treatments only ranges up to 43%.

Despite this level of success of interferon alpha therapy *in vivo*, treatment with interferons has no effect in cell lines that are supporting HDV replication [8], [9], [10]. In fact, a recent report indicated that HDV replication can interfere with the ability of the cell to respond to interferon [11]. Here we have confirmed and extended the understanding of how HDV RNA replication might interfere with responsiveness. Our findings include evidence that HDV RNA replication in cell lines can be modestly inhibited if interferon expression is induced. We have also made use of primary human hepatocytes that are susceptible to infection by HDV. With such a system, we find that treatments with interferons alpha or gamma, have a significant effect especially when applied around the time of initiation of HDV replication. In summary, we here resolve what has been an apparent disagreement between in vivo and in vitro studies, and hopefully clear the way for rational improvements in therapies for chronic HDV infections.

## Results

As described above, previous studies with transfected cell lines indicate that HDV RNA accumulation is resistant to the application of interferon treatments. In addition, HDV RNA accumulation might actually interfere with the cellular response to interferon. To address this latter possibility we made use of two cell lines derived from 293 cells, a human embryonic kidney derived cell line. 293-δAg cells contain a single DNA copy of the HDV small antigen, with expression under TET control [12]. By 24 h after TET induction, millions of copies of δAg accumulate per cell, without toxicity or significant effect on expression of cellular genes [12], [13]. This cell line was transfected with HDV RNA to produce the 293-HDV cell line. After 24 h of TET induction, these cells accumulate not only δAg but also ∼40,000 copies of HDV genomic RNA, and a smaller amount of antigenomic HDV RNA. As described by Guo et al. [14] we induced an interferon response in these two cell lines by transfection with poly(IC) at 6 h before TET induction. The response was measured after 24 h of TET induction, by assaying for interferon-beta1 mRNA.

**Figure 1:**
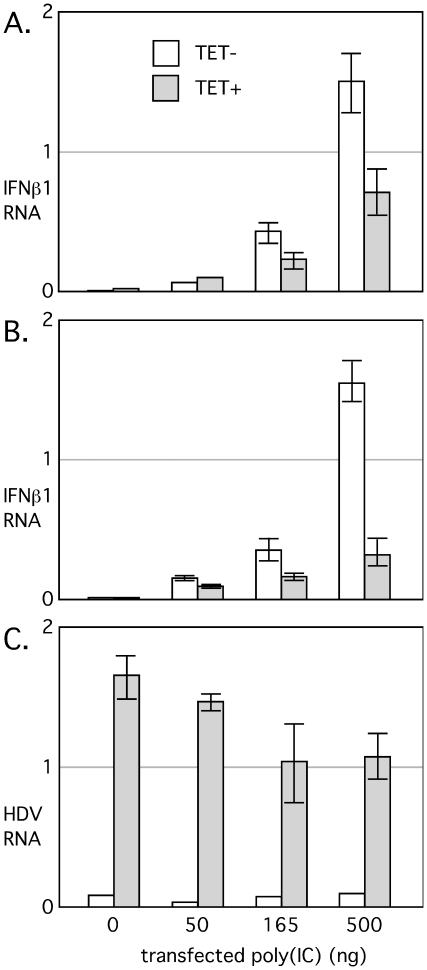
Transfection of poly(IC) into 293-δAg and 293-HDV cells, with and without TET induction. Panel A: 293-δAg cells in a 24-well format were transfected with the indicated amounts of poly(IC) and after 6 h induced with or without TET. At 24 h total RNA was extracted and assayed by realtime PCR for interferon-beta1. Panels B and C: As in A, but using 293-HDV cells. In B and C the assays are for interferon-beta1 and HDV RNA, respectively. The experiment was performed in triplicate. Values are expressed in arbitrary units along with the standard error of the mean which includes the errors in both the control and the treated cultures.

As shown in Fig. 1A, transfection of 293-δAg cells with increasing amounts of poly(IC) produced a robust induction of interferon-beta1 mRNA in the absence of TET induction of δAg synthesis. When δAg was induced by TET interferon-beta1 mRNA accumulation was reduced 2-fold. As shown in Fig. 1B for 293-HDV cells, HDV RNA accumulation reduced interferon beta1 induction by an additional 2-fold.

Our findings are thus is in agreement with the report of Pugnale et al. [11] that HDV can suppress innate immunity, albeit incompletely. In addition, as shown in Fig. 1C, there was a modest effect, associated with the poly(IC) transfection, on the ability of the induced cells to accumulate HDV RNA.

Some caution is nevertheless needed in interpreting this inhibition of interferon induction since we have previously shown for 293-HDV cells, using whole genome mRNA array analysis, that TET induction alone, in the absence of poly(IC), is sufficient to produce in 24 h greater than 2-fold changes, increases or decreases, in more than 1,000 host mRNA [13]. And, these were not in genes associated with the innate immune response.

We next turned our attention to whether the application of interferons alpha and gamma could affect the ability of primary human hepatocytes to support HDV infection [15]. In this system, HDV is not secreted since HBV is not present, but HDV genome replication, which is not HBV-dependent, still takes place. Thus, we can study the effect of these interferons in the absence any competition with HBV, and in the absence of virus assembly and spread. Interferons alpha and gamma were applied at 600 units/ml. We estimated this would be the theoretical maximum therapeutic concentration of interferon alpha in a patient injected with 3 million units in a blood volume of 5 liters.

**Figure 2:**
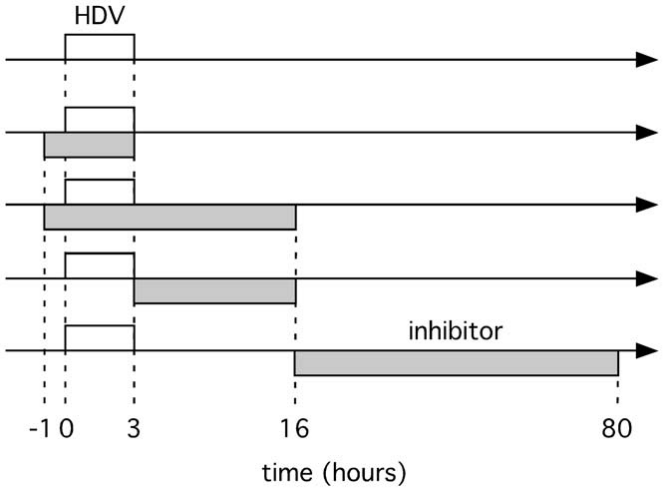
Representation of time-lines for exposure of primary hepatocytes to HDV and potential inhibitors. For the five time-lines shown the open box indicates the period of HDV exposure and the shaded box the exposure to inhibitor. The effects on HDV replication of such treatments with three different inhibitors are summarized in Table 1.

Our experimental procedure was to expose the hepatocytes to virus for only 3 h. The potential inhibitors were applied at different times relative to this. HDV RNA levels were measured 6 days post-infection, using realtime PCR. The time-lines of such treatments with virus and inhibitors are represented in Fig. 2, and the results are summarized in Table 1. It can be seen that significant inhibition was obtained when either interferon was applied during the period of virus exposure but less so after 16 h, when virus replication was initiated.

**Table 1:** HDV replication is inhibited by application of interferons alpha or gamma.

	% HDV replication in presence of inhibitor		
Time when inhibitor present (hours)	PreS1 peptide	IFN-alpha	IFN-gamma
−1 to +3	9±6	19±5	20±12
−1 to +16	4±2	8±2	15±10
+3 to +16	9±6	17±8	38±21
+16 to +80	65±36	37±17	40±23

Times of addition of inhibitors are measured relative to 3-h of virus exposure. The preS1 peptide was present at 50 nM while the interferons were at 600 U/ml. All assays were performed in at least triplicate, and the data expressed relative to control hepatocytes that were not treated with inhibitors. The indicated standard error of the mean includes the errors in both the control and the treated cultures.

As a control for these studies we used a synthetic peptide that blocks HDV infection by inhibiting virus entry into hepatocytes but has no effect on viral genome replication in infected hepatocytes. Previous studies by Urban and others have shown that the N-terminal region of the large envelope protein of HBV is necessary for infectivity of HBV, and also of HDV [16], [17], [18], [19]. Furthermore, chemically synthesized forms of this region give strong inhibition of infection both with cultured cells and even in vivo, in mice transplanted with human hepatocytes [20]. The chemically synthesized peptide, referred to as preS1 peptide, is 47 amino acids long with an N-terminal myristoylation and was used at 50 nM. As shown in Table 1 this peptide strongly inhibited HDV infection when present during the 3 h of exposure to virus. However, when added beyond 16 h, and even for a much longer period (64 h), it had little effect. Similar to the preS1 peptide, the primary effect of applying interferons alpha and gamma occurred at the time of infection (Table 1). And, consistent with the studies in cell lines, interferon application had less effect once the hepatocytes were infected (Table 1).

**Figure 3:**
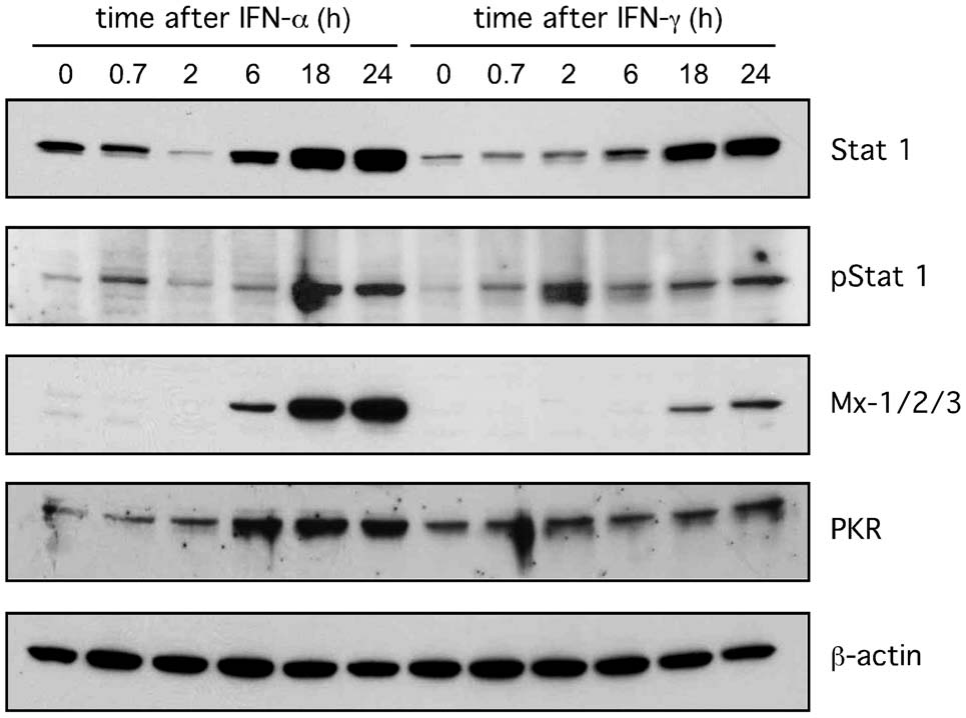
Time-dependent induction by interferons alpha and gamma of innate immune response proteins in primary hepatocytes. Hepatocytes were exposed to either interferons alpha or gamma at 600 units/ml. At time points out to 24 h, as indicated, total cell protein was extracted and analyzed by immunoblot to detect the indicated host proteins. Note that after 2 hours of treatment with interferon gamma, the pSTAT1 increases but not the total STAT1; this is because only a small fraction of the total STAT1 undergoes phosphorylation.

Interferon treatments can induce an innate response but this outcome can be host cell specific [21], [22]. Therefore, it was obligatory to test whether that our experimental conditions induced such a response in the cultured primary human hepatocytes. Using immunoblot assays we found, as shown in Fig. 3, that both interferons at 600 units/ml induced time-dependent changes of known host innate response proteins in the primary hepatocytes. Increases were seen for Stat 1, pStat 1, Mx-1/2/3 and PKR. Interferon alpha had more effect than interferon gamma. As expected no changes were detected for beta-actin.

**Figure 4:**
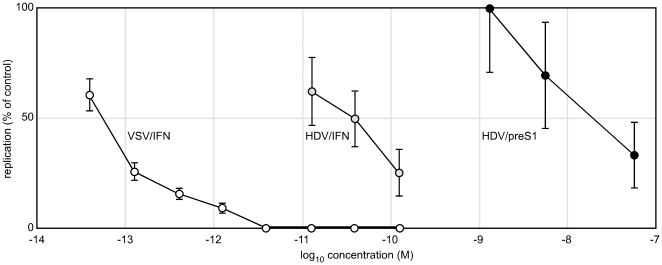
Dose response of HDV and VSV infections to interferon-alpha and preS1 peptide. For HDV infections the inhibitors were present from 24 h prior to infection and during the 16 h of exposure to virus. Replication was assayed at 6 days post-infection by realtime PCR, as in Fig. 1C. The assays were performed in triplicate, and average values are expressed as a percentage relative to the untreated controls, with the indicated standard error of the mean. For the VSV infections, the interferon was only present during the 16 h of virus exposure. VSV replication was assayed at 16 h by counting GFP positive cells. The values are expressed relative to untreated controls and the errors indicate the standard deviation as the square root of the mean. Interferon-alpha concentrations are expressed here as molarities, with 600 units/ml = 0.12 nM. The preS1 peptide, like interferon-alpha, can inhibit HDV infection, but the needed molarity is 300-times more. Interferon-alpha can inhibit VSV infection but the concentration is 1,000-time less than that needed to inhibit HDV.

Because this induction of proteins associated with host immunity was greatest after 24 h of treatment (Fig. 3) we next asked if pretreatment with interferon enhanced the inhibition of HDV replication. At the same time we performed a dose response curve for interferon alpha and the preS1 peptide. The results are summarized in Fig. 4. The extended pretreatment did not increase the action of interferon alpha or the preS1 peptide. This result further supports the interpretation that inhibition is largely achieved during and/or soon after virus entry. For the preS1 peptide we expect the inhibition to be during rather than after virus entry.

Note that we calculated that 600 units/ml of recombinant interferon alpha is equivalent to 0.12 nM. Thus while preS1 peptide is inhibitory, on a molar basis it is about 300-times less active than the recombinant interferon alpha.

We also compared the sensitivity of VSV infection of primary hepatocytes to interferon alpha. For this we used a modified VSV that expresses GFP. VSV not only replicates faster than HDV but also will release new virus that can spread to uninfected cells. Therefore, we reduced the multiplicity of infection to about 0.01, and limited the VSV exposure of hepatocytes to 16 h, without and with increasing doses of interferon. At 16 h post-infection GFP positive cells were counted. As shown in Fig. 4, the action of interferon alpha was potent with 1 pM (6 units/ml) giving >90% inhibition, and ≥3 pM (20 units/ml) giving >99.5% inhibition. Thus inhibition of VSV in primary hepatocytes is achieved at molar concentrations of interferon about 1,000 times lower than required for HDV.

## Discussion

Studies by others have shown that efficient inhibition of VSV replication by interferon is multi-faceted and related to induction of a host innate response [23] just as we confirmed here for VSV and primary human hepatocytes (Figs. 3 and 4). The observed inhibition of HDV replication was real but less efficient, requiring much higher molar concentrations of interferon (Fig. 4). This efficiency was not increased by the addition of a prior interferon treatment (Table 1 and Fig. 4). And, since there was less inhibition when the interferon was added for a much longer period but after the initiation of infection (Table 1), we suggest that for HDV infections, interferons act primarily during the process of virus entry. Our interpretation could explain why previous in vitro studies that used HDV transfection rather than infection, demonstrated little antiviral activity of interferon treatment. Additional support for our explanation is as follows. In previous studies we and others have found that HDV entry into susceptible cells is a slow process [18], [24]. Of the virus that attaches to cells within a 3-h period of exposure, the majority requires as much as 16 h more before virus entry is achieved. Such data have been obtained using as inhibitor the preS1 peptide synthesized and characterized by Urban and coworkers [18], [19]. It acts after virus attachment, in the slow but as yet unexplained process of virus entry [18]. In the present studies interferons were like preS1 peptide in terms of the dependence of the achieved effects on the time of inhibitor exposure relative to the period of virus exposure (Table 1). Thus we assert that interferons are largely acting in the same way. There are other agents that act predominantly at HDV entry into primary human hepatocytes. These include suramin, BBG, PPADS, and to a lesser extent heparin [24]. On a molar basis, these agents are much less active than the preS1 peptide, which in turn is much less active than the interferons (Fig. 4). However, as a unifying hypothesis, we suggest they all act largely by perturbations of a slow multi-step process by which virus entry is achieved.

At the same time we also understood that under certain situations, interferons can affect HDV replication at steps other than virus entry. In an earlier in vitro study, we failed to detect an effect of interferon treatments on inducible HDV RNA synthesis and accumulation. In this earlier study using 293-HDV cells there was no of evidence of a host response to the interferon [8]. However, here we document that following transfection with poly(IC), there is an induction of interferon beta1 and a modest 2-fold effect on HDV RNA accumulation (Fig. 1B). We also tested the converse situation and observed that HDV RNA accumulation interfered with the poly(IC) response (Fig. 1B). It did have a 4-fold effect, and in this we agree with the studies of Pugnale et al. [11]. However, we also found that expression of δAg alone, in the absence of HDV RNA accumulation, was sufficient for a 2-fold effect (Fig. 1A).

Pugnale et al. did find that after HDV replication was induced by transfection of liver cell lines, there was inhibition of the ability of interferon to induce host response genes [11]. As part of a strategy for additional testing of this in vitro phenomenon, we used HDV infection to achieve infection of >1% of all cultured hepatocytes. Then at 5 days after HDV exposure, we determined whether cells could still be infected within 16 h by VSV and whether 20 units/ml of interferon present during this 16 h would inhibit the VSV. If HDV replication were to specifically suppress the interferon response, then the VSV would now replicate in the HDV infected cells. However, by immunostaining for δAg and GFP, we found that VSV was only able to infect cells not infected by HDV, and that the VSV infection was inhibited by the interferon application (data not shown). Thus, prior HDV infection did not specifically suppress the ability of cells to respond to interferon, and thereby allow interferon-resistant VSV replication. More likely, we propose that 5 days of HDV replication rendered the cells resistant to VSV infection.

The present studies show that application of interferons can significantly inhibit HDV entry and/or initiation of replication in susceptible primary human hepatocytes. This study did not assay possible additional late effects of interferons, such as on HDV RNA-editing and HBV-dependent assembly of progeny HDV particles.

The in vitro studies presented here rationalize the use of interferons for in vivo therapies of chronic HDV, even with the use of the highest tolerable doses. There still needs to be a search for new strategies, possibly as combinations therapies, which could improve treatment for chronic HDV. Maybe exogenous interferons can be replaced by immunomodulators such as IMO-2125, which is a synthetic agonist of the toll-like receptor, TLR-9 [25]. The preS1 peptide is active in vitro and in vivo and has unique advantages relative to interferons [20]. Farnesylation inhibitors that act against HDV assembly have been tested in model systems [26]. And maybe there is a role for siRNA that target the mRNA for the essential small delta antigen [27].

## Materials and Methods

### Materials

Recombinant human interferon alpha 2b (Intron A) was from Scherring and gamma were from Pestka Biological Laboratories. Chemically synthesized HBV preS1 peptide was a gift of Stephan Urban.

### Cells and virus

We have previously described the derivation from human embryonic kidney cells (HEK293 [28]) of two lines with tetracycline (TET) inducible HDV sequences [12]. 293-δAg is induced to express the small form of the HDV antigen, which is essential for RNA accumulation. 293-HDV is derived from this cell line, and contains transfected copies of HDV RNA that replicate and increase in number when δAg is induced. For the experiments of Fig. 1, these two cell lines were transfected with poly(IC) (InvivoGen) for 6 h prior to a 24 h TET induction. Not induced cells served as a control. Total RNA was extracted and assayed by realtime PCR for interferon-beta1 and HDV RNA. For HDV infection studies virus was assembled as previously described [15]. Briefly, Huh7 cells [29] were transfected with two plasmids, one to express the HBV envelope proteins and another to initiate HDV genome replication. Media was collected between days 6 and 13. After clarification, virus was precipitated by the addition of polyethylene glycol to 10%, collected by centrifugation, and resuspended in 1% of the original volume of media. Primary human hepatocytes (PHH) in a 48-well format, were purchased from either Cellzdirect or Celsis, and maintained in the recommended hepatocyte growth medium. After exposure of cells to virus, the media was removed and the cells washed two times. The cells were harvested at 6 days post-infection. Vesicular stomatitis virus (VSV) carrying a gene for green fluorescent protein (GFP) was from the lab of Glenn Barber [30]. Hepatocyte cultures were infected with VSV at a titer sufficient to infect 1% of cells, in the absence or presence of recombinant interferons, for a period of 16 h, after which infection was assayed by counting GFP-positive cells.

### RNA extraction and realtime PCR

To assay HDV replication total RNA was extracted and realtime PCR was performed after DNase treatments, as previously described [24]. Assays for interferon beta-1 mRNA used primers purchased from Applied Biosystems.

### Protein extraction and immunoblot assays

Individual wells of PHH were exposed to interferons alpha or gamma for periods of up to 24 h. The wells were then washed with phosphate buffered saline and lysed in buffer containing TX100 and phosphatase inhibitors. Finally, samples were sonicated before addition of Laemmli buffer and immunoblot analysis, as previously described [31]. Chemi-luminescence assays were performed using primary antibodies: Stat-1 from Becton-Dickinson, phosphoStat-1 from Upstate, beta-actin from Sigma, Mx1/2/3 and PKR B10 from Santa Cruz.
